# A novel murine model of combined hepatocellular carcinoma and intrahepatic cholangiocarcinoma

**DOI:** 10.1186/s12967-022-03791-z

**Published:** 2022-12-09

**Authors:** Ru-Chen Xu, Fu Wang, Jia-Lei Sun, Weinire Abuduwaili, Guang-Cong Zhang, Zhi-Yong Liu, Tao-Tao Liu, Ling Dong, Xi-Zhong Shen, Ji-Min Zhu

**Affiliations:** 1 Department of Gastroenterology and Hepatology, Zhongshan Hospital of Fudan University, 180 Fenglin Rd., Shanghai, 200032 China; 2 Shanghai Institute of Liver Diseases, Shanghai, China; 3 Key Laboratory of Medical Molecular Virology, Shanghai Medical College of Fudan University, Shanghai, China

**Keywords:** Myc, AKT1, p53, LAMB1, Sleeping Beauty-dependent transposon system, In situ electroporation

## Abstract

Primary liver cancer (PLC) is a common gastrointestinal malignancy worldwide. While hepatocellular carcinoma (HCC) and intrahepatic cholangiocarcinoma (ICC) are two major pathologic types of PLC, combined HCC and ICC (cHCC-ICC) is a relatively rare subtype that shares both hepatocyte and cholangiocyte differentiation. However, the molecular feature of this unique tumor remains elusive because of its low incidence and lack of a suitable animal model. Herein, we generated a novel spontaneous cHCC-ICC model using a Sleeping Beauty-dependent transposon plasmid co-expressing oncogenic *Myc* and *AKT1* and a CRISPR-Cas9 plasmid expressing single-guide RNA targeting *p53* into mouse hepatocytes via in situ electroporation. The histological and transcriptional analysis confirmed that this model exhibits cHCC-ICC features and activates pathways committing cHCC-ICC formation, such as TGF-β, WNT, and NF-κB. Using this model, we further screened and identified LAMB1, a protein involved in cell adhesion and migration, as a potential therapeutic target for cHCC-ICC. In conclusion, our work presents a novel genetic cHCC-ICC model and provides new insights into cHCC-ICC.

**Supplementary Information:**

The online version contains supplementary material available at 10.1186/s12967-022-03791-z.

## Introduction

Primary liver cancer (PLC), including hepatocellular carcinoma (HCC) and intrahepatic cholangiocarcinoma (ICC), represents the third cause of cancer-related death [1, 2]. Research has revealed that ICC and HCC share a monoclonal origin with bidirectional phenotype differentiation and may appear simultaneously [3]. Combined HCC and ICC (cHCC-ICC) is a rare tumor accounting for 0.4–14.2% of all PLC [4–7]. Since the diagnosis of cHCC-ICC relies on evidence of histological findings and patients who are not suitable for resection may be misdiagnosed with HCC or ICC alone, the actual incidence underestimates the current cHCC-ICC burden [5]. Although curative surgical resection or liver transplantation is considered the mainstay of clinical practice, up to 80% of patients relapse within 5 years due to lymph node metastasis and vascular invasion [8, 9]. Unfortunately, the 5-year survival in patients with unresectable cHCC-ICC does not exceed 30% due to inadequate response to current treatments [10, 11]. Taking these findings into account when making treatment decisions, a more focused understanding of the molecular pathology and identification of potential therapeutic targets of cHCC-ICC thus are urgently needed.

Compared to xenograft tumor models, genetically engineered tumor models develop *de novo* tumors that closely imitate the histopathological features of their human counterparts [12]. Hydrodynamic tail vein injection (HDVI) and in situ electroporation (Epo) are two ways to transfer foreign plasmid DNA directly into hepatocytes. HDVI can create a pressurized blood force that redirects the blood flow directly into the liver, leading to plasmid DNA entering the intracellular compartment of hepatocytes [13], while electroporation is an efficient way to introduce foreign genes into cultured cells and able to in situ transfer plasmids into hepatocytes [14].

Recently, high-throughput genomic studies have revealed a series of driver genes contributing to HCC or ICC tumorigenesis [15, 16]. *c-Myc* and *TP53* are two top frequently mutated genes in HCC patients. Recently, co-delivery of *c-Myc*-encoding plasmid and CRISPR/Cas9-mediated *p53* knockout via HDVI successfully developed spontaneous HCC in mice [17, 18]. In another study, Seehawer M et al. constructed a vector that co-expressed *Myc* and *AKT1* to establish HCC in *p19Arf*^*−/−*^ mice via HDVI [19]. Interestingly, the same vector led to ICC tumorigenesis by the approach of Epo, which could cause in situ necroptosis microenvironment, highlighting the hepatic microenvironment may contribute to lineage commitment during tumorigenesis [19].

In the current study, we applied a Sleeping Beauty-dependent transposon plasmid co-expressing oncogenic *Myc* and *AKT1* in combination with a CRISPR-Cas9 plasmid expressing single-guide RNA targeting *p53* to compare their tumorigenic capacity via either HDVI- or Epo-dependent hepatocyte delivery. Notably, we found transfection of these plasmids by Epo led to the cHCC-ICC formation. Taking advantage of this novel spontaneous model, we proposed that LAMB1 may serve as a therapeutic target for cHCC-ICC.

## Materials and methods

### Vectors

A plasmid that co-expressing oncogenic Myc and AKT1 was a kind gift and has been constructed and described previously [19]. The SB13 transposase-encoding vector was kindly provided by Dr Yue Zhao. pX330 backbone expressing sgRNA targeting p53 was obtained from Tyler Jacks (Addgene plasmid #59910).

### Animal studies

Male 4 to 6-week-old C57BL/6 J mice were purchased from Charles River (Shanghai, China), and all the animals used in the study were fed in a specific pathogen-free facility. All animal care and experimental protocols were approved by the Institutional Animal Care and Use Committee (IACUC) of Zhongshan Hospital, Fudan University.

### Hydrodynamic tail vein injection and in situ electroporation

For hydrodynamic tail vein injection, 30 µg *Myc* + *AKT1* co-expressing plasmid, 30 µg pX330 sg-p53 plasmid, and 10 µg SB13 transposase-encoding plasmid were prepared in 2 ml of sterile PBS and injected into a tail vein within 3–5 s per mouse. For in situ electroporation, 6-week-old wild-type C57BL/6 J mice were anesthetized, and the right lateral liver lobe was exposed after midline laparotomy. Plasmids described above were resolved in 50 µg sterile PBS and injected into the right lateral liver lobe using an insulin needle. In situ electroporation was performed with Squaure Wave Electroporator (Nepa Gene). The voltage and duration of electric pulse were 70 V and 75 ms, respectively. Two pulses were applied, and the interval was 500 ms.

### Immunohistochemistry

Immunohistochemistry (IHC) was performed as previously described [20]. In brief, the sections of tumors were incubated with the following antibodies: HNF4α (ab201460, Abcam), CK-19 (ab52625, Abcam), PCNA (2586, Cell Signaling Technology), p-AKT (Ser473, 4060, Cell Signaling Technology), p-ERK (Thr202/Tyr204, 4370, Cell Signaling Technology), p-NF-κB (Ser536, 3033, Cell Signaling Technology), β-catenin (8480, Cell Signaling Technology), TGF-β (21898-1-AP, Proteintech) and FGFR2 (13042-1-AP, Proteintech).

### RNA-sequencing and analysis

RNA-sequencing for whole transcriptome analysis was performed using Illumina NovaSeq 6000 platform according to the manufacturer’s protocol. Three biological replicates were applied for RNA-seq, and all RNA samples passed quality control with 5–8 Gb and Q20 ≥ 90. Hierarchical clustering of RNA-seq was performed using R language with the ‘pheatmap’ package. A Euclidean method was used to calculate distance measurements, while the ‘complete’ method was utilized to calculate the dissimilarity values for hierarchical clustering. For the heatmap visualization, gene expression values were normalized.

### Bioinformatic analysis

Gene set enrichment analysis (GSEA) was performed using GSEA software version 4.1.0 with 1,000 permutations of the gene sets. The FPKM values from the RNA-seq were compared against the specific gene sets. Gene sets used in this study were downloaded from the MSigdb database (http://software.broadinstitute.org/gsea/msigdb/index.jsp).

### Statistics

The statistical analysis was performed using Prism Graphpad 7.0 software. Quantitative variables were analyzed by paired *t*-test. Kaplan-Meier analysis was used to compare OS between HDVI and Epo groups. Data were presented as mean ± standard deviation. *p* < 0.05 was considered statistically significant.

## Results

### Generation of spontaneous liver cancer model through HDVI and Epo

**Fig. 1:**
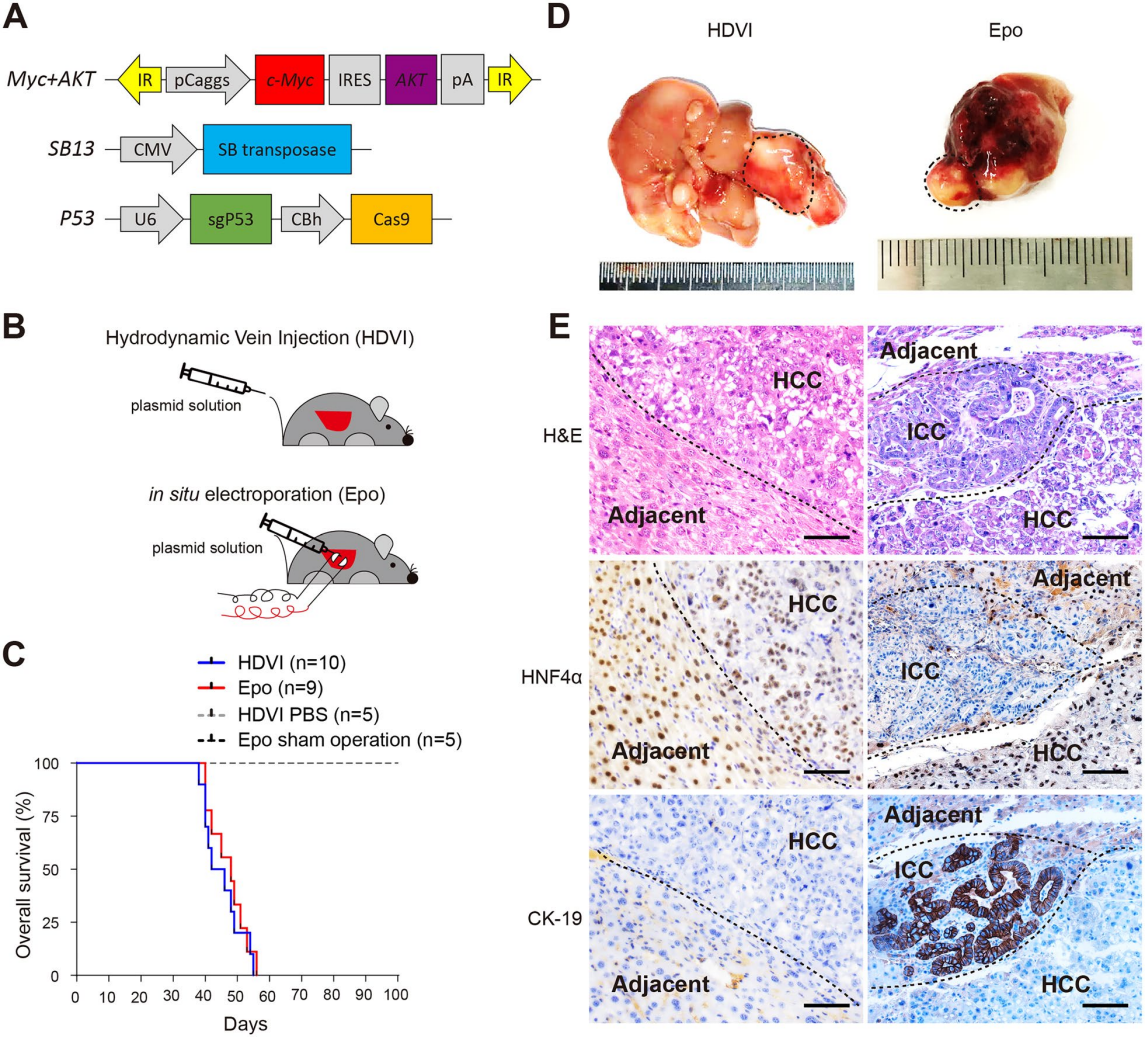
Generation of spontaneous primary liver cancer model using hydrodynamic tail vein injection (HDVI) and in situ electroporation (Epo). **A** Schematic of plasmids designed. **B** Schematic of intrahepatic delivery of plasmids through HDVI or Epo. **C** The overall survival rate of *MAPHDVI* and *MAPEpo* mice, respectively. **D** Representative images of livers from mice received HDVI- or Epo-dependent plasmids via intrahepatic transfection. **E** Representative hematoxylin and eosin (H&E), HNF4α, and CK-19 staining of liver tissues shown in Fig. 1D. Scale bar, 200 μm

*Myc*, *AKT1*, and *TP53* are among the top frequent mutated genes in PLC. To assess their roles in liver cancer tumorigenesis, a vector expressing constitutively active oncogenic *Myc* and *AKT1*, and single-guild RNA targeting *Tp53* (sgTp53) was transfected into mouse liver cells based on a Sleeping Beauty (SB) transposon system via either HDVI or Epo (Fig. 1A, B). Both methods induced liver cancer 3–4 weeks after vector delivery, showing comparable overall survival rates (Fig. 1C). Tumors generated by HDVI (termed as *MAPHDVI*) exhibited multifocal neoplasia, while tumors generated by Epo (termed as *MAPEpo*) exhibited a unilocular pattern (Fig. 1D). H&E staining revealed disorderly and mitotically activated epithelial cells with abnormal pleomorphic nuclei and loss of polarity in all *MAPHDVI* tumors (Fig. 1E). Also, bile duct-like tissues were observed in *MAPEpo* tumors rather than in *MAPHDVI* tumors (Fig. 1E). IHC staining confirmed that *MAPHDVI* were HCC, as evidenced by solid nuclear staining for hepatocyte nuclear factor 4-alpha (HNF4α), a liver-specific marker, but negative staining for cytokeratin 19 (CK-19), a marker of biliary differentiation. Interestingly, staining of both HNF4α and CK-19 in *MAPEpo* tumors with a clear boundary confirmed a combined HCC and ICC within the same tumor (Fig. 1E). These data indicate that induction of *Myc* and *AKT1* and loss of *TP53* by HDVI lead to spontaneous HCC formation while Epo leads to cHCC-ICC.

### Transcriptional analysis of the *MAPHDVI* and *MAPEpo* tumors

**Fig. 2:**
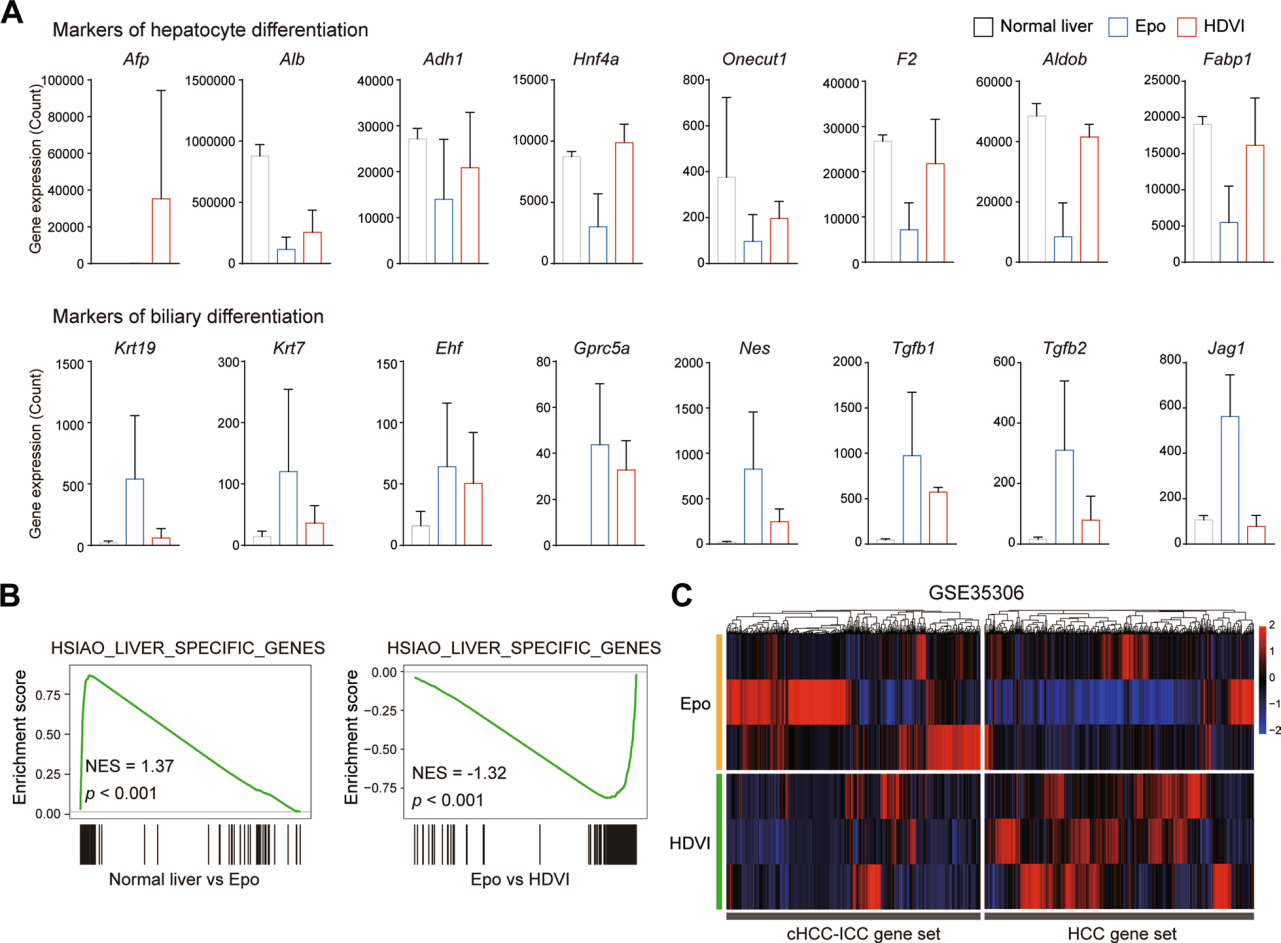
Transcriptome analysis of *MAP*^*HDVI*^ and *MAP*^*Epo*^ tumors. **A** Expression of genes that involves in hepatocyte and biliary differentiation. **B** GESA analysis using transcriptome profiles of *MAP*^*HDVI*^ and *MAP*^*Epo*^ tumors. Enrichment plots were presented for liver-specific gene signature. **C** Hierarchal clustering analysis of HCC- and cHCC-ICC–related genes was performed in *MAP*^*HDVI*^ and *MAP*^*Epo*^ tumors

To further validate the morphological findings, RNA-seq analysis for *MAPHDVI* and *MAPEpo* was performed to investigate the transcriptome differences. Tumor tissues from *MAPHDVI* and *MAPEpo*, along with normal liver tissue from wild-type C57BL/6 J mice as control, were firstly assessed for hepatocyte and biliary differentiation markers. The results suggested that *MAPHDVI* tumors exhibited higher expression of hepatic markers, including *Afp*, *Alb*, *Adh1*, *Hnf4a*, *Onecut1*, *F2*, *Aldob*, and *Fabp1*. However, genes involved in biliary differentiation, including *Krt19*, *Krt7*, *Ehf*, *Gprc5a*, *Nes*, *Tgfb1*, *Tgfb2*, and *Jag1*, were highly expressed in *MAPEpo* tumors but not in *MAPHDVI* tumors [21] (Fig. 2A). Further GESA analysis confirmed that liver-specific gene signature was significantly enriched in normal liver tissues and *MAPHDVI* tumors rather than in *MAPEpo* tumors (Fig. 1B). A previous study transcriptionally profiled 20 cHCC-ICC and successfully identified a series of gene sets that could represent cHCC-ICC and HCC genome features [22]. cHCC-ICC-related gene set comprised 588 up-regulated genes, while the HCC-related gene set contained 656 up-regulated genes. Using these gene sets, Hierarchical clustering analysis revealed that *MAPEpo* tumors had higher expression of genes associated with cHCC-ICC. In contrast, *MAPHDVI* tumors had higher expression of genes involved in HCC. Collectively, these transcriptome data further support the successful generation of spontaneous cHCC-ICC through Epo.

### Signaling pathways that commit cHCC-ICC activates in *MAPEpo* tumors

**Fig. 3:**
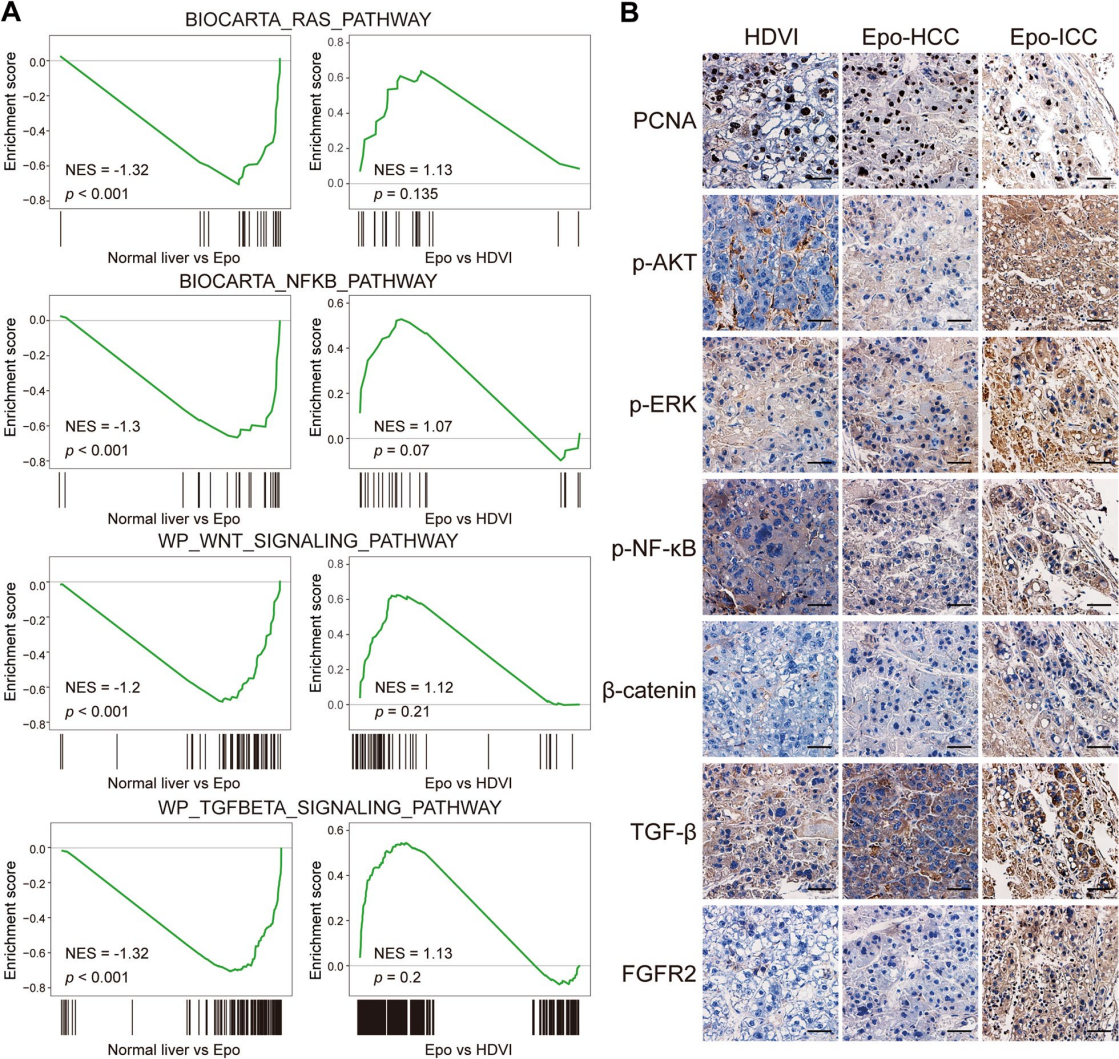
Signaling pathways activated in *MAP*^*HDVI*^ and *MAP*^*Epo*^ tumors. **A** GESA analysis of gene signatures of indicated pathways in normal liver tissues, *MAP*^*HDVI*^ and *MAP*^*Epo*^ tumors. **B** Representative images of PCNA, p-AKT, p-ERK, p-NFκB, β-catenin, TGF-β, and FGFR2 staining in *MAP*^*HDVI*^ tumors and HCC or ICC component of *MAP*^*Epo*^ tumors, respectively. Scale bars, 50 μm

Several key signaling pathways, including the AKT, RAS, NFκB, WNT-β-catenin, and TGF-β signaling pathways, have been identified as responsible for initiating and developing cHCC-ICC [23]. We, therefore, examined whether these pathways were also activated in the *MAPEpo* model. GESA analysis proved that genes in the RAS, NF-κB, WNT, and TGF-β signaling pathways were significantly enriched in *MAPEpo* tumors rather than in normal liver tissues (Fig. 3A). Although the enrichment score produced no statistical discrepancy, these pathways tended to be relatively enriched in *MAPEpo* tumor rather than in *MAPHDVI* tumor, probably because of the mixed HCC and ICC within the tumor (Fig. 3A). IHC analysis for HCC and ICC components was subsequently performed in *MAPEpo* tumors to dissect this phenotype better. A comparable PCNA staining pattern among *MAPHDVI*, HCC-*MAPEpo*, and ICC-*MAPEpo* tumors was observed, suggesting a similar proliferation in these tumor cells (Fig. 3B). Notably, IHC staining was strongly positive for p-AKT, p-ERK, p–NF-κB, β-catenin, and TGF-β in the ICC component of the *MAPEpo* tumors, where FGFR2 served as the positive control (Fig. 3B). Taken together, these data suggest that the signaling pathways that contributed to cHCC-ICC formation and development are activated in *MAPEpo* tumors.

### Identification of LAMB1 may serve as a potential therapy target for cHCC-ICC

**Fig. 4:**
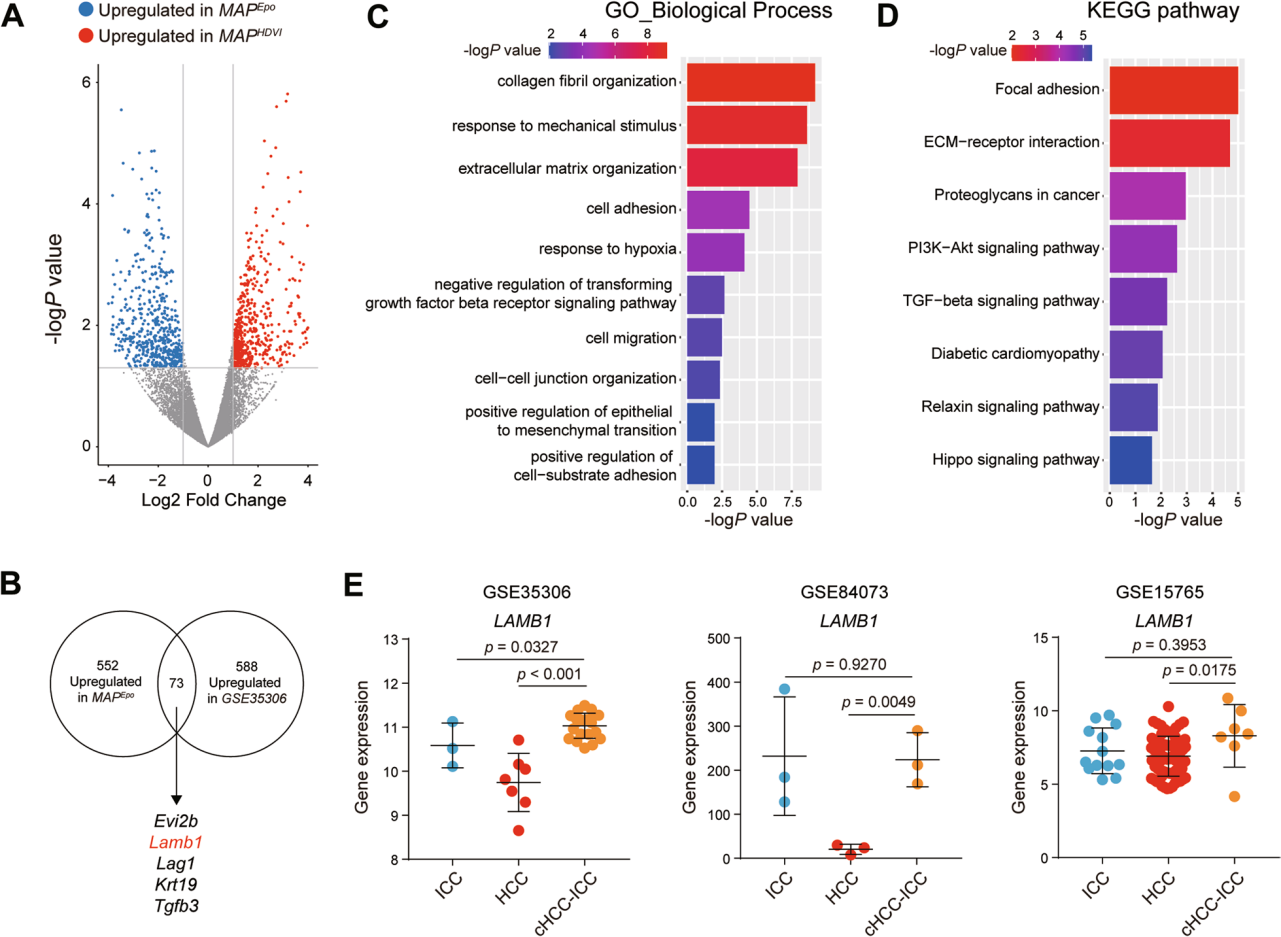
*LAMB1* was upregulated in cHCC-ICC. **A** Volcano plot analysis of 1120 differential genes expressed between *MAP*^*HDVI*^ and *MAP*^*Epo*^ tumors. The criteria of analysis were Log_2_FC ≥ 1 or ≤ -1, *p* < 0.05. Genes that upregulated in *MAP*^*Epo*^ tumors were shown in blue plots, while genes that upregulated in *MAP*^*HDVI*^ tumors were in red plots. **B** Venn gram presented 73 overlapping genes between 552 upregulated genes in *MAP*^*Epo*^ tumors and 588 upregulated genes in the GSE35306 data set. **C** GO and **D** KEGG analysis of 73 genes obtained in Fig. 4B. **E** The expression of *LAMB1* gene in human ICC, HCC, and cHCC-ICC from GSE35306, GSE84073, and GSE15765 datasets

As our preliminary data implied that *MAPEpo* tumor resembled human cHCC-ICC, we, therefore, utilized this model to explore its clinical relevance. Differentially expressed genes (Log2FC ≥ 1 or ≤ − 1, *p* < 0.05) between *MAPEpo* tumor and *MAPHDVI* tumor were profiled to find genes involved in cHCC-ICC but not in HCC. The results indicated that 552 genes were overexpressed while 568 genes were downregulated in *MAPEpo* tumor compared to that in *MAPHDVI* (Fig. 4A). To further verify the consistency of our findings, previously reported upregulated genes in cHCC-ICC and upregulated genes in *MAPEpo* tumors were compared. A total of 73 overlapping genes were identified (Fig. 4B).

Using these overlapping genes as input, Gene Oncology analysis revealed that biological processes (BP) were mainly enriched in tumor metastasis-related changes such as extracellular matrix organization (ECM), cell adhesion, and cell migration (Fig. 4C). Kyoto Encyclopedia of Genes and Genomes (KEGG) analysis demonstrated that focal adhesion and ECM-receptor interaction were among the top two pathways enriched (Fig. 4D). These results were consistent with the invasive feature of cHCC-ICC.

In the 73 overlapping genes, *Lamb1*, which contributes to ECM activity and cell adhesion and is highly expressed in cHCC-ICC, was identified. We also found that *LAMB1* expression in human cHCC-ICC (GSE35306) was significantly higher than in both ICC and HCC tissues. For further validation, we analyzed another two published datasets (GSE84073 and GSE15765) that contain transcriptome data of human ICC, HCC, and cHCC-ICC. It also exhibited that *LAMB1* was upregulated in human cHCC-ICC compared with HCC (Fig. 4E). Considering minimal data on cHCC-ICC are available, no significant changes of *Lamb1* were observed between cHCC-ICC and ICC. To sum up, these findings imply that *Lamb1* may serve as a therapeutic target for cHCC-ICC, and further study is needed to dissect its role in cHCC-ICC.

## Discussion

cHCC-ICC is a rare type of PLC but has attracted increasing attention in recent years. Because the diagnosis of cHCC-ICC largely depends on histochemistry, the true incidence of cHCC-ICC is likely to be underestimated, making the knowledge and management of cHCC-ICC inaccessible. Thus, a tumor animal model that resembles clinical cHCC-ICC is essential for further investigation. In this study, we generated a spontaneous cHCC-ICC by induction of oncogenic *Myc* and *AKT1* and loss of *p53* through in situ electroporation. Evidence showed that this model shared similar transcriptome and oncogenic signaling pathways with human cHCC-ICC. Most importantly, we, for the first time, identified LAMB1 as a potential therapy target for cHCC-ICC.

Most solid tumors, especially HCC, develop in the context of chronic diseases and are composed of heterogeneous malignant cells. Importantly, the heterogeneous feature of tumor cells is a key reason for clinical drug resistance. Although traditional syngeneic or xenograft models are easy to perform, these models are unable to fully mimic specific human disease conditions and neglect the heterogeneous feature of tumor tissues. Thus, these common models have a limit to evaluating the drugs pre-clinically. It is well known that tumor cells are transformed from normal cells carrying oncogenic mutations, making it possible to induce PLC by genetic engineering. Accumulative studies have revealed specific mutations that lead to either HCC or ICC tumorigenesis. For example, *c-Myc*, *CTNNB1* and *TP53* are among top mutated genes for HCC patients [24]; while *KRAS*, a powerful oncogene involved in glandular malignant, is able to induce ICC when specifically expressed in mouse hepatocytes [25]. Currently, genetic engineered mouse model (GEMM) is an ideal tool for study HCC or ICC tumorigenesis and has clear genetic background resembling human disease, making it suitable for pre-clinical estimation. Unfortunately, few studies focus on cHCC-ICC as it’s not entirely clear how cHCC-ICC occurs. A recent study proved that an inflammatory tumor microenvironment directs lineage commitment of the PLC [19]. Here, we used electroporation to induce a necroptotic liver microenvironment and established cHCC-ICC formation by combing *Myc* and *AKT1* knockin with *p53* knockout. The morphological and genetic evidences proved successful induction of cHCC-ICC. Our work provides a simple GEMM for cHCC-ICC with direct clinical translational value. However, our study also has limitations. It remains elusive whether other gene combinations can lead to the cHCC-ICC formation in the same experimental setting. Moreover, our model does not exhibit the metastatic feature of cHCC-ICC, as evidenced by no lung metastatic lesion observed (data not shown). New gene combinations that can lead to metastasis should be tested in the future.

Whether cHCC-ICC is a unique or a subtype of HCC or ICC has long been controversial. cHCC-ICC can be further divided into three subtypes according to Allen and Lisa’s criteria that are separate type (HCC and ICC components physically separated), combined type (HCC and ICC component in the same tumor with clear boundaries), and mixed type (HCC and ICC component in the same tumor with no boundaries) [26]. Histologically, we found that *MAPEpo* tumors exhibited features with a combined type of cHCC-ICC. Recently, Xue et al. [3] comprehensively analyzed a total of 133 cHCC-ICC cases and revealed that combined type of cHCC-ICC acquired intense ICC-like landscapes, including high expression of *KRT19* but a lower expression of HCC markers (including *AFP* and *GPC3*), which is also supported by our transcriptome data (Fig. 2A). Compared with *MAPHDVI* tumors, *MAPEpo* tumors tended to express markers of biliary differentiation, especially for *Krt19*, *Krt7*, *Nes*, *Tgfb2*, and *Jag1*. Consistently, *Afp* expression is much higher in *MAPHDVI* tumors than in *MAPEpo* tumors. These data suggested that *MAPEpo* tumors might exhibit more ICC-like characteristics.

Despite apparent molecular discrepancy among subtypes of cHCC-ICC, they all have poorer prognosis and more invasive features than HCC and are similar to ICC [27]. Interactions between cell adhesion or migration and ECM are vital factors that mediate tumor metastasis. In our study, we identified 73 overlapping genes that are highly upregulated in cHCC-ICC. Bioinformatic analysis indicated that these genes were mainly involved in ECM bioprocesses and cell adhesion. These findings further reinforced the invasive feature of cHCC-ICC.

Laminins, a family of extracellular matrix glycoproteins, are among the predominant component of ECM [28]. Evidence reported that laminins participated in tumor metastasis by promoting cell adhesion and migration, and their receptors expressed on the tumor cell surface [29–31]. We screened these 73 genes and identified a main differential gene *Lamb1*, a member of the laminin family. Taking advantage of several data sets reported previously, we established that *Lamb1* was upregulated in cHCC-ICC compared to HCC, but its expression seemed comparable with ICC. It has been reported that LAMB1 overexpressed in several types of tumors and correlated with tumor metastasis and poor prognosis [32–34]. The prognosis value of LAMB1 was further screened tin TCGA database. The results suggested that LAMB1 is elevated in a series of tumors, but LAMB1 is negatively correlated with both OS and DFS for LIHC, CHOL, and COAD (Additional file 1: Fig. S1). Most importantly, KEGG and GSEA analysis showed that LAMB1 played a pivotal role in pro-metastatic processes, including focal adhesion, ECM-receptor interaction, and cellular junction in LIHC, CHOL, and COAD, highlighting that LAMB1 might also be a potential treating target for cHCC-ICC (Additional file 1: Fig. S2). Since clinical transcriptome data of patients with cHCC-ICC remains very limited, we were able only to verify the expression of *Lamb1* in current published data; further larger-scale studies should be performed to assess its clinical value. Summarily, the present study established a human-resembling cHCC-ICC model via in situ electroporation. This novel preclinical model can be used to investigate the molecular feature of cHCC-ICC.

## Supplementary Information

**Additional file 1. Figure S1.** Multi-cancer analysis of the expression and prognostic role of LAMB1. **A** LAMB1 is significantly upregulated in multiple cancer types from TCGA data. **B** Kaplan-Meier analysis of the association between LAMB1 expression and OS or DFS in LIHC, CHOL, and COAD datasets. **Figure S2.** Multi-cancer analysis of biological functions and significant pathway of LAMB1. **A** Top 20 pathways enriched in the KEGG analysis in LIHC, CHOL, and COAD datasets. The red box represents pro-metastatic processes, including focal adhesion, ECM-receptor interaction, and cellular junction. **B** GESA analysis of gene signatures of indicated pathways in LIHC, CHOL, and COAD datasets.

## Data Availability

The datasets used and analyzed during the current study are available from the corresponding author on reasonable request.
