# Supplementary material for: A novel murine model of combined hepatocellular carcinoma and intrahepatic cholangiocarcinoma
Source: J Transl Med. 2022 Dec 9;20:579. doi: 10.1186/s12967-022-03791-z (PMC9733131; doi:10.1186/s12967-022-03791-z)
Supplement: Supplementary file 1 — Additional file 1. Figure S1. Multi-cancer analysis of the expression and prognostic role of LAMB1. A LAMB1 is significantly upregulated in multiple cancer types from TCGA data. B Kaplan-Meier analysis of the association between LAMB1 expression and OS or DFS in LIHC, CHOL, and COAD datasets. Figure S2. Multi-cancer analysis of biological functions and significant pathway of LAMB1. A Top 20 pathways enriched in the KEGG analysis in LIHC, CHOL, and COAD datasets. The red box represents pro-metastatic processes, including focal adhesion, ECM-receptor interaction, and cellular junction. B GESA analysis of gene signatures of indicated pathways in LIHC, CHOL, and COAD datasets. [file 12967_2022_3791_MOESM1_ESM.pdf]

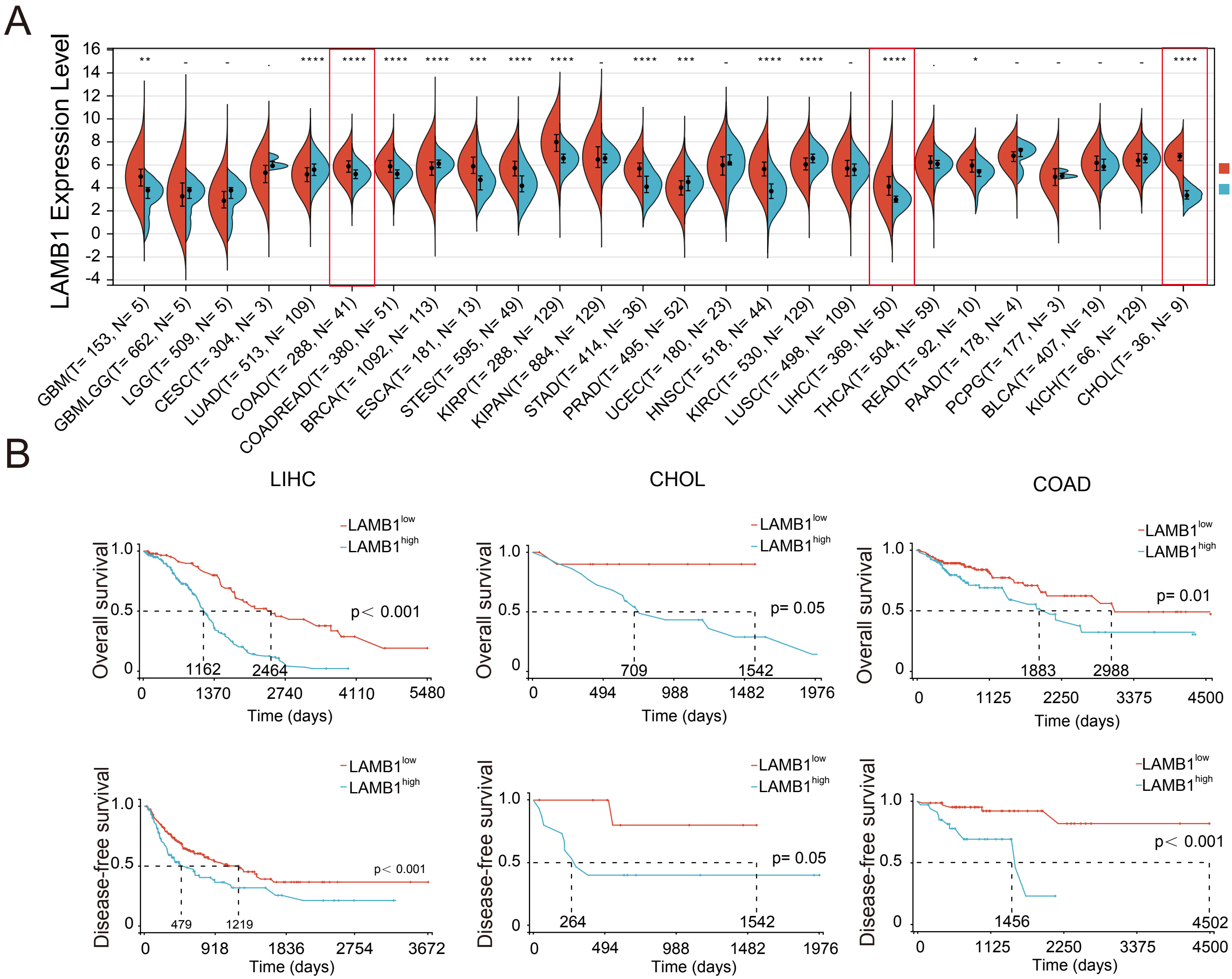

Supplementary Figure 1. **Multi-cancer analysis of the expression and prognostic role of LAMB1.** (A) LAMB1 is significantly upregulated in multiple cancer types from TCGA data. (B) Kaplan-Meier analysis of the association between LAMB1 expression and OS or DFS in LIHC, CHOL, and COAD datasets.

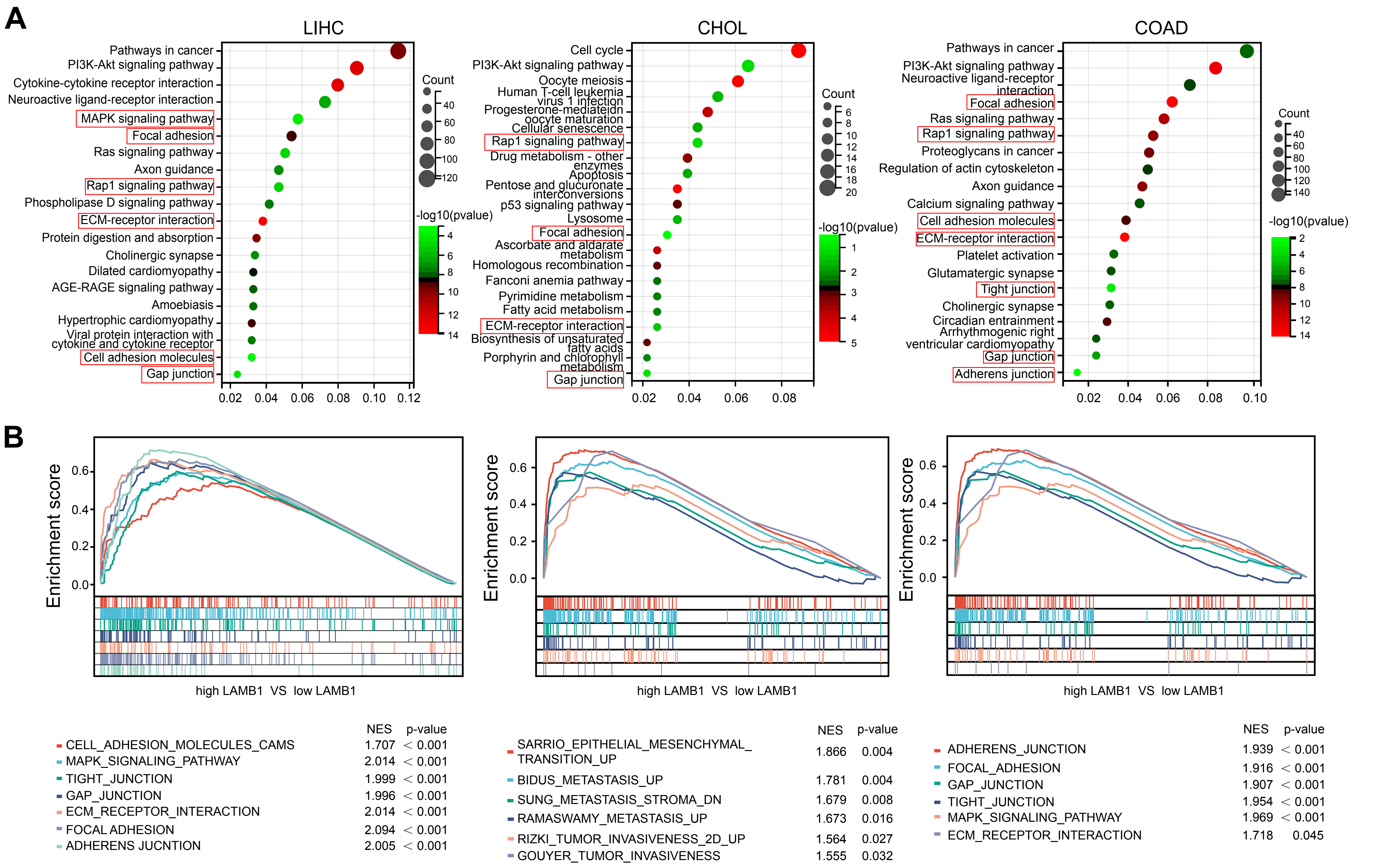

Supplementary Figure 2 **Multi-cancer analysis of biological functions and significant pathway of LAMB1.** (A) Top 20 pathways enriched in the KEGG analysis in LIHC, CHOL, and COAD datasets. The red box represents pro-metastatic processes, including focal adhesion, ECM-receptor interaction, and cellular junction. (B) GESA analysis of gene signatures of indicated pathways in LIHC, CHOL, and COAD datasets.
